# Supplementary material for: Hydrogen Attenuates Inflammation by Inducing Early M2 Macrophage Polarization in Skin Wound Healing
Source: Pharmaceuticals (Basel). 2023 Jun 15;16(6):885. doi: 10.3390/ph16060885 (PMC10302845; doi:10.3390/ph16060885)
Supplement: Supplementary file 1 [file pharmaceuticals-16-00885-s001.zip › Supplementary Fig1 .pdf]

Supplemental Information

Hydrogen Attenuates Inflammation by Inducing Early M2 Macrophage Polarization in Skin Wound Healing

Pengxiang Zhao [1,2,3], Zisong Cai [1,2,3], Xujuan Zhang [1,2,3], Mengyu Liu [1,2,3], Fei Xie [1,2,3], Ziyi Liu [1,2,3], Shidong Lu [1,2,3] and Xuemei Ma [1,2,3,*]

1 Faculty of Environment and Life, Beijing University of Technology, Beijing 100124, China
2 Beijing Molecular Hydrogen Research Center, Beijing 100124, China
3 Beijing International Science and Technology Cooperation Base of Antivirus Drug, Beijing 100124, China
* Correspondence: xmma@bjut.edu.cn; Tel.: +86-136-6118-8049

Results

H_2 inhibited the systemic inflammatory response

Skin defects may cause systemic inflammatory responses, so we performed routine blood tests to determine the number of leukocytes and the percentages of neutrophils, lymphocytes, and monocytes in the blood of each group on the first three days (Fig. 4). The number of leukocytes in the blood corresponds to the state of systemic inflammation of the organism. From day 1 to day 3, no significant difference was observed in Leukocyte counts in all groups (Fig. 4A). Neutrophils remove cellular debris and release cytokines to create conditions for subsequent repair. The proportion of neutrophils in the H_2 group gradually decreased from 24 h to 72 h; at 48 h, the proportion of neutrophils was higher in the H_2 and NAC groups than in the control group ($P < 0.01$ between the NAC group and the control group), while at 72 h, the proportion of neutrophils in the H_2 group was lower than in the control group (Fig. 4B). The proportion of lymphocytes in the NAC group was significantly lower than in the control group ($P < 0.01$) and no significant difference was observed compared with the H_2 group at 48 h; at 72 h, the proportion of lymphocytes was higher in the H_2 group than in the other two groups (Fig. 3C), and no significant differences observed. At 72 h, monocyte counts in the H_2 group were lower than those in the control groups ($P < 0.052$). Therefore, taking insight into the blood cell count results, systemic inflammatory responses in the H_2 group have no obvious changes at the first 72 h, while the monocytes number in H_2 group was slightly decreased on the 72 h.

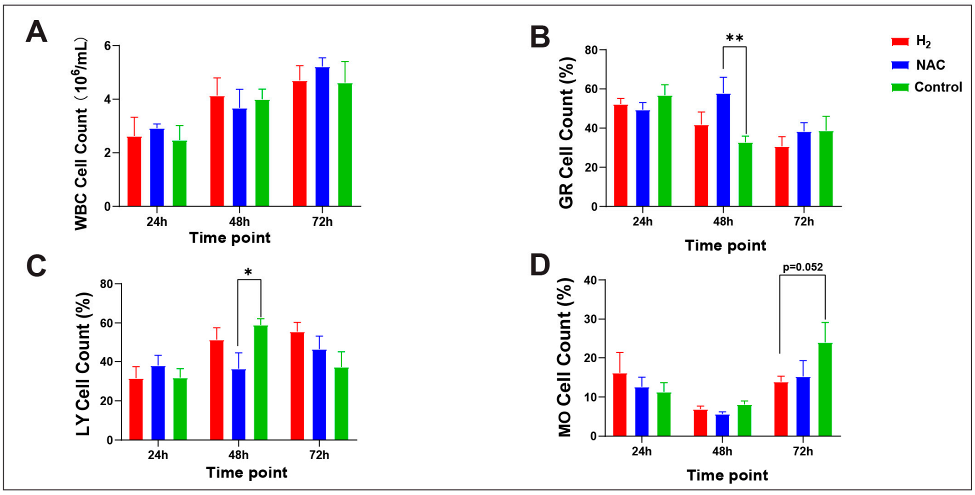

Figure S1. Routine blood tests during the inflammatory stages of wound healing. Routine blood tests of the three groups. **(A)** White blood cell (WBC) count. **(B)** Granulocytes (GR). **(C)** Lymphocytes (LY). **(D)** Monocytes (MO). Percentages in whole blood at 24, 48, and 72 h after wounding. Data were analyzed by two-way ANOVA and plotted as mean±SEM. *$P < 0.05$; **$P < 0.01$; ***$P < 0.001$.
